# The oxidative/anti-oxidative effects of sevoflurane on reproductive system of females: An experimental study

**DOI:** 10.4274/tjod.78871

**Published:** 2017-12-30

**Authors:** Hatice Yılmaz Doğru, İsmail Benli, Serkan Doğru

**Affiliations:** 1 Gaziosmanpaşa University Faculty of Medicine, Department of Obstetrics and Gynecology, Tokat, Turkey; 2 Gaziosmanpaşa University Faculty of Medicine, Department of Biochemistry, Tokat, Turkey; 3 Gaziosmanpaşa University Faculty of Medicine, Department of Anesthesiology and Reanimation, Tokat, Turkey

**Keywords:** Oxidative stress, nitric oxide, malondialdehyde, reproductive system

## Abstract

**Objective:**

A permanent balance exists between the production and elimination of reactive oxygen species in all living organisms. The aim of this study was to evaluate the effects of sevoflurane possibly causing an imbalance in the equation of reactive oxygen species on the female rat reproductive system.

**Materials and Methods:**

A total of 30 adult female Wistar-albino rats were placed into an anesthesia chamber to administer sevoflurane. Rats were randomly divided into six groups, each group consisting of five rats: the control group received 2 L/min O_2_ 18 min/day for seven days; the first group received 1 minimum alveolar concentration (MAC) of sevoflurane and 2 L/min O_2_ 18 min/day for seven days; the second group received 1 MAC of sevoflurane and 2 L/min O_2_ 18 min/day for seven days with no treatment for the next seven days; the third group received 1 MAC of sevoflurane and 2 L/min O_2_ 18 min/day for 14 days; the fourth group received 1 MAC of sevoflurane and 2 L/min O_2_ 18 min/day for 14 days with no treatment for the next seven days; and the fifth group received 1 MAC of sevoflurane and 2 L/min O_2_ 18 min/day for 14 days with no treatment for the next 14 days. Bilateral ovaries were subsequently removed for biochemical analysis of tissue anti-oxidative enzyme levels.

**Results:**

Slight fluctuations were detected in mean nitric oxide, prostaglandin E2, prostaglandin F2-alpha, superoxide dismutase, glutathione peroxidase, malondialdehyde, alginate dialdehyde, and xanthine oxidase levels between the groups; however, the differences were not significant (p>0.05).

**Conclusion:**

Sevoflurane has no effect on the activity of anti-oxidant systems in the rat ovary.

## PRECIS:

Sevoflurane did not show any effect on the activity of anti-oxidant systems in the rat ovary.

## INTRODUCTION

Sevoflurane [2,2,2-trifluoro-1- (trifluoromethyl) ethyl fluoromethyl ether], which has a boiling point of 58.6 °C, a vapor pressure of 160 mm hemoglobin at 20 °C, and a blood-gas partition coefficient of 0.69 (approximately half of isoflurane), is pleasant-smelling and relatively non-irritating to the airways providing a high inhaled concentration without any adverse effects or irritation^(1)^.

Oxidative stress is a condition as a consequence of an irregularity between the production and elimination of reactive oxygen species that are spontaneously generated during aerobic respiration and consumed endogenously. The tendency of the balance through free oxygen radicals can be deleterious for the sustainability of the life of a cell. Nitric oxide, one of the free radicals, plays a crucial role in the female reproduction system and manages the endometrial, myometrial, and microvasculatory tasks by paracrine functions.

Superoxide dismutase activity has been shown in the granulose and theca cells of the follicle, where glutathione peroxidase enzyme is localized in follicular fluid^(2)^. In contrast, with alginate dialdehyde, the oxidized form of alginate, there has been no study investigating the possible effects on female reproduction^(3)^.

Furthermore, prostaglandin E2 and F2-alpha are autocrine and paracrine lipid mediators that are increased during the late secretory phase in which reactive oxygen species trigger the production of prostaglandin F2-alpha(2,3). A study conducted by Yalçınkaya et al.^(4)^ on the effects of follicular fluid nitric oxide, malondialdehyde, and reduced glutathione on in vitro fertilization outcomes demonstrated that malondialdehyde level was high in the follicular fluid of women with pregnancy, whereas nitric oxide was low. They also found that a positive correlation existed between malondialdehyde levels and the number of grade 1 embryos, and fertilization rates^(4)^. In this context, several drugs have been investigated for the production or the effects on the anti-oxidative enzyme systems of the body. The role of anesthetic agents is a very popular topic for researchers. Various studies conducted on the impacts of volatile anesthetics on the anti-oxidant system of different tissues showed controversial results. Sevoflurane decreases the intensity of oxidative stress and induced the activity of antioxidant defense mechanisms in erythrocytes^(5,6,7,8,9,10)^. Limited studies have been performed on the impacts of sevoflurane on reproductive tissues^(8,9)^. Therefore, the present study aimed to evaluate the impact of sevoflurane on the oxidant/anti-oxidant systems in the female reproductive tissue of rats.

## MATERIALS AND METHODS

After approval of the Animal Experiments Local Ethics Committee (2016-HADYEK-12), a total of 30 adult female Wistar-albino rats (90 days-old, 250-300 grams, all selected in the same period of estrus cycle as estrus by assessing vaginal smears) were obtained from the experimental medicine unit. Rats were housed in a room sustained at 20-24 °C with a 12-h light-dark cycle (lights on at 06:00 to 18:00) and constant humidity of 40-50%. All animals were kept in polycarbonate cages and given tap water ad libitum.

For sevoflurane exposure, rats were moved to a 40x50x60 cm anesthesia chamber, which had a connection with an anesthesia system (Prima SP Alpha, Penlon Limited, Oxon, UK). As previously described by Ceyhan et al.^(11)^, two holes, one at the top left side of the chamber and the other at the upper right side of the chamber, were opened for anesthetic gas inlet and outlet. Animals were randomly separated into six groups, each group included five rats: the control group (C) was administered 2 L/min O_2_ 18 min/day for seven days; the first group (S1) was administered 1 minimum alveolar concentration (MAC) of sevoflurane and 2 L/min O_2_ 18 min/day for seven days; the second group (S2) was administered 1 MAC of sevoflurane and 2 L/min O_2_ 18 min/day for seven days with no treatment for the next seven days; the third group (S3) was administered 1 MAC of sevoflurane and 2 L/min O_2_ 18 min/day for 14 days; the fourth group (S4) was administered 1 MAC of sevoflurane and 2 L/min O_2_ in 18 min/day for 14 days with no treatment for the next seven days; and the fifth group (S5) was administered 1 MAC of sevoflurane and 2 L/min O_2_ 18 min/day for 14 days with no treatment for the next 14 days. Animals were anesthetized by intraperitoneal injection of ketamine 90 mg/kg (Alfasan International B.V., Woerden, NL) with xylazine 10 mg/kg (Alfasan International B.V., Woerden, NL), and were killed by performing a cervical dislocation at the end of the 7^th^ day in C and S1, the 14^th^ day in S2 and S3, the 2^nd^ day in S4, and the 28^th^ day in S5. Bilateral ovaries were subsequently removed. The ovaries of each animal were placed on ice and then transferred to a -70 °C freezer where they remained frozen until biochemical analysis of tissue anti-oxidative enzyme levels.

### Tissue nitric oxide level detection

Nitric oxide is a fast-eliminated molecule that is oxidized to nitrite and subsequently nitrate, which are used as the index parameters of nitric oxide production. The Griess reaction was performed for the detection of plasma nitrite and nitrate levels^(12)^. First, the protein fraction of the samples was removed using Somogyi reagent. After the total transformation of nitrite to nitrate using coppered cadmium granules, nitrite levels were calculated using spectrophotometry at 545 nm. A reaction curve was constructed with a pack of serial dilutions (10^-8^-10^-3^ mol/L) of sodium nitrate. Outcomes were calculated as micromole per liter (micromol/L).

### Tissue superoxide dismutase activity detection

Total (copper-zinc, manganese) superoxide dismutase activity was defined using the method previously described by Sun et al.^(13)^ In brief, the method depends on the formation of nitro blue tetrazolium chloride reduction through the xanthine-xanthine oxidase system. After adding 1 mL ethanol/chloroform mixture (5/3, v/v) to the sample and centrifugation, superoxide dismutase activity was evaluated in the ethanol phase of the sample. One unit of superoxide dismutase was determined as the enzyme intensity providing 50% inhibition in the nitro blue tetrazolium chloride reduction ratio. Superoxide dismutase activity was displayed as units per liter (U/L).

### Tissue glutathione peroxidase activity detection

Glutathione peroxidase activity was calculated using the technique of Paglia and Valentine^(14)^. A chemical reaction was triggered by adding hydrogen peroxide in the mixture of sodium azide, nicotinamide adenine dinucleotide phosphate, glutathione reductase, and reduced glutathione. Thereafter, the absorbance of this solution was calculated using a spectrophotometer at 340 nm. Activity is presented as units per milliliter (U/mL).

### Tissue xanthine oxidase activity determination

The measurement was performed using the method reported by Prajda and Weber.^(15)^. The activity of xanthine oxidase was calculated using the production of uric acid via xanthine through a spectrophotometry elevation at 293 nm. A functional curve was established using 10-50 mL concentrations of standard xanthine oxidase solutions (Sigma X-1875). One unit of activity was determined as one micromol of uric acid produced per minute (37 °C, pH 7.5), and presented as U/mL.

### Tissue alginate dialdehyde activity detection

Tissue protein concentrations were measured using the technique previously described by Lowry et al.^(16)^.

### Prostaglandin E2 concentration

Prostaglandin E2 levels were measured using a prostaglandin E2 (514010) enzyme-linked immunosorbent assay (ELISA) (Cayman Chemical Company, Ann Arbor, MI, USA) kit as described in the manufacturer’s instructions and presented as pg/mL.

### Prostaglandin F2-alpha concentration

Prostaglandin F2-alpha levels were calculated using a prostaglandin F2-alpha (516011) ELISA (Cayman Chemical Company, Ann Arbor, MI, USA) kit as described in the manufacturer’s instructions and presented as pg/mL.

### Statistical Analysis

Normality and variance were analyzed using the One-Sample Kolmogorov-Smirnov test. Numeric data are presented as means and standard deviation, and categorical data as frequency and percentage. Oxidative stress marker levels were analyzed using the One-Way ANOVA test, and post-hoc comparisons were conducted using Tukey’s honest significant difference test. The Statistical Package for Social Sciences (SPSS Inc., Chicago, IL) version 20.0 program was used to complete all analyses. P values <0.05 were accepted as statistically significant in all analyses.

## RESULTS

All animals completed the experiment. No death or any complications occurred during the experimental exposure period. Slight fluctuations were detected in mean nitric oxide, prostaglandin E2, prostaglandin F2-alpha, superoxide dismutase, glutathione peroxidase, malondialdehyde, alginate dialdehyde, and xanthine oxidase levels; however, the differences were not significant (p>0.05, Table 1). The minimum levels of nitric oxide, prostaglandin E2, prostaglandin F2-alpha, superoxide dismutase, glutathione peroxidase, malondialdehyde, alginate dialdehyde, and xanthine oxidase were 0.75±0.07 micromol/L in S4, 501.63±149.29 pg/mL in S2, 0.11±0.01 U/mg in S4, 2.51±0.26 U/g in S1, 1.85±0.83 nmol/g in C, 55.38±29.09 nmol/g in S4, and 0.78±0.23 U/g in S5, respectively.

## DISCUSSION

The effect of reactive oxygen species appears to be a double-edged sword, they are used as signaling factors in physiologic conditions but also have additional roles in pathologic conditions including in the female reproductive system. There is continuous stability between oxidants and antioxidants. Superoxide dismutase, copper-zinc superoxide dismutase, and manganese superoxide dismutase are located in the granulosa and theca cells of developing follicles^(17)^. In addition, glutathione peroxidase activity can be seen in follicular fluid^(18)^. In contrast to the detrimental effects, oxidative stress may be one of the main factors that can manage ovarian germ and stromal cell physiology. Vascular changes and proteolytic cascades are the major factors regulating ovulation. The signaling between these two processes are established by vascular endothelial growth factor, reactive oxygen, reactive nitrogen species, and cytokines^(17,18)^. Ben-Shlomo et al.^(18)^ showed that interleukin 1-alpha caused nitric oxide accumulation in rat ovaries, suggesting a possible interaction between cytokines and reactive nitric oxide species. Oxidative stress and cytokines are demonstrated as intercellular and intracellular messengers in rat ovarian tissues^(19,20)^. There is an evident balance between antioxidant enzymes and reactive oxygen species in the ovaries. Superoxide dismutase, one of the antioxidant enzymes, is intensely present in the theca interna cells of antral follicles. An experimental study revealed that luteal cells reduced the expression of estradiol and progesterone hormones after adding hydrogen peroxide (reactive oxygen species) to the cell culture environment^(21)^. The preovulatory follicle is heavily guarded against oxidative stress in which glutathione peroxidase is the major enzyme maintaining its lower hydroperoxide levels thus holds a crucial role in gametogenesis and fertilization^(22)^. In this context, the reactive oxygen scavenging system has an important role in all organ systems and the reproductive system. Environmental factors such as anesthetic agents may alter the fine balance of this regulatory mechanism.

The present study showed that acute exposure to sevoflurane by inhalation did not demonstrate any evident differences on oxidative stress markers of reproductive tissues in female rats. Various oxidative enzymes including copper-zinc superoxide dismutase, manganese superoxide dismutase, glutathione peroxidase, gamma-glutamyl-cysteine synthase, and catalase have an important role to protect the oocyte against the effects of oxidative damage during maturation and early preimplantation embryo development. Studies showed that codes for superoxide dismutase were ready to transcript in oocytes at all stages of maturation^(23)^. Oxidative damage may occur with several drugs and diseases, and shows a similar route associated with their development^(24)^. Various studies have been conducted on oxidative stress and inflammation, mainly focusing on isoflurane and sevoflurane^(25)^. Despite the limited data about the effects of sevoflurane on female ovary tissues, a study by Türkan et al.^(26)^ on liver, kidney, brain, and lung of rats demonstrated that sevoflurane caused an increase only in the activity of anti-oxidative enzyme, malondialdehyde in lungs. Allaouchiche et al.^(8)^ evaluated the impacts of sevoflurane and desflurane on lungs in mechanically-ventilated swine. Thereafter, they analyzed bronchoalveolar lavage fluid specimens and blood samples for levels of superoxide dismutase, glutathione peroxidase, and malondialdehyde. They found that sevoflurane led to an evident raise in malondialdehyde levels in both bronchoalveolar lavage fluid and plasma.

Free oxygen radicals have crucial roles in the normal immune defense system and metabolic activity. In contrast, the overproduction or disrupted elimination of these radicals may cause mild-to-severe cellular damage and DNA mutations by chemical modifications of cellular protein, carbohydrate, lipid, and nucleotides. Oxidative stress is described as the incline in enzymes of the anti-oxidant defense system^(27)^. Anesthetic agents may have a direct effect on the anti-oxidant system causing a decrease in the blood flow of the liver, thus leading to a relative increase in the magnitude of free oxygen radical production^(28)^. In contrast, with minor fluctuations detected in oxidative stress markers in the present study, sevoflurane showed no apparent disturbances in the anti-oxidative system of testicular tissue.

### Study Limitation

Due to the financial aspects of experimental studies, we could not enhance the design of the study by including neither ovarian cell DNA nor immunohistochemical analysis. These kind of techniques could have provided detailed information about the effects of sevoflurane on the ovarian cell.

## CONCLUSION

This is the first study to define the impacts of sevoflurane on the female rat reproductive system using oxidative system biomarkers. Our findings revealed that sevoflurane has no effect on the activity of anti-oxidant systems in the rat ovary. This result suggests that sevoflurane is a safe anesthetic agent in reproduction-aged females. Comprehensive studies are needed to confirm this outcome.

## Figures and Tables

**Table 1:**
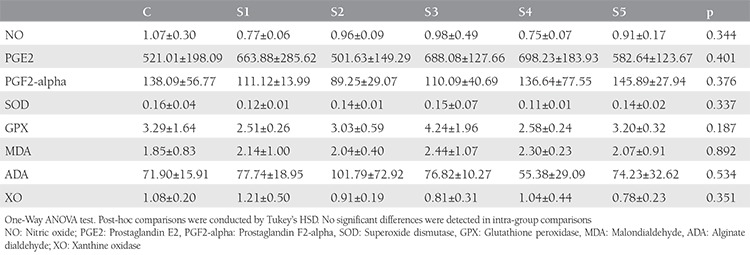
Oxidative stress marker levels in rats exposed to sevoflurane
